# Determinant Factors in the Production of a Co-Occluded Binary Mixture of *Helicoverpa armigera* Alphabaculovirus (HearNPV) Genotypes with Desirable Insecticidal Characteristics

**DOI:** 10.1371/journal.pone.0164486

**Published:** 2016-10-12

**Authors:** Maite Arrizubieta, Oihane Simón, Trevor Williams, Primitivo Caballero

**Affiliations:** 1 Bioinsecticidas Microbianos, Instituto de Agrobiotecnología, CSIC-UPNA, Gobierno de Navarra, 31192 Mutilva Baja, Navarra, Spain; 2 Instituto de Ecología AC, Xalapa, Veracruz 91070, Mexico; 3 Laboratorio de Entomología Agrícola y Patología de Insectos, Departamento de Producción Agraria, Universidad Pública de Navarra, 31006 Pamplona, Navarra, Spain; Institute of Plant Physiology and Ecology Shanghai Institutes for Biological Sciences, CHINA

## Abstract

A co-occluded binary mixture of *Helicoverpa armigera* nucleopolyhedrovirus genotypes HearSP1B and HearLB6 at a 1:1 ratio (HearSP1B+HearLB6) was selected for the development of a virus-based biological insecticide, which requires an efficient large-scale production system. *In vivo* production systems require optimization studies in each host-virus pathosystem. In the present study, the effects of larval instar, rearing density, timing of inoculation, inoculum concentration and temperature on the production of HearSP1B+HearLB6 in its homologous host were evaluated. The high prevalence of cannibalism in infected larvae (40–87%) indicated that insects require individual rearing to avoid major losses in OB production. The OB production of recently molted fifth instars (7.0 x 10^9^ OBs/larva), combined with a high prevalence of mortality (85.7%), resulted in the highest overall OB yield (6.0 x 10^11^ OBs/100 inoculated larvae), compared to those of third or fourth instars. However, as inoculum concentration did not influence final OB yield, the lowest concentration, LC_80_ (5.5 x 10^6^ OBs/ml), was selected. Incubation temperature did not significantly influence OB yield, although larvae maintained at 30°C died 13 and 34 hours earlier than those incubated at 26°C and 23°C, respectively. We conclude that the efficient production of HearSP1B+HearLB6 OBs involves inoculation of recently molted fifth instars with a LC_80_ concentration of OBs followed by individual rearing at 30°C.

## Introduction

The cotton bollworm, *Helicoverpa armigera* (Hübner) (Lepidoptera: Noctuidae), is a major polyphagous insect pest in a wide range of crops in many parts of the world [1, 2]. Originally native to the Old World, this pest has been introduced to Asia and Oceania, and has recently invaded Brazil and Argentina [3]. Its recent appearance in Puerto Rico now represents a serious threat to the United States, Mexico, Central America and the islands of the Caribbean [4].

Spain is a major producer of tomatoes for export to European countries and elsewhere, with an area of over 49,000 hectares (ha) in production that yields over 4.0 million tonnes per year [5]. *H*. *armigera* is the most important pest in field-grown tomato crops in this region [6]. Infestations of *H*. *armigera* are controlled by applying synthetic broad-spectrum insecticides and, more recently, using newer biorational products such as *Bacillus thuringiensis*. However, repeated use of these products has led to the appearance of insecticide-resistant pest biotypes [7]. Moreover, in order to comply with European Union regulations, the presence of pesticide residues in products from this region are carefully monitored [8]. This has highlighted the need to develop effective alternative control methods of pest control that are economically-viable and which do not contribute to the presence of xenobiotic residues in food produce [6, 7, 9].

The *H*. *armigera* single nucleopolyhedrovirus (HearNPV) (genus *Alphabaculovirus*, family Baculoviridae) has proved effective as the basis for biological insecticide products targeted at this pest [10, 11]. In previous studies conducted in our laboratory, a binary mixture of HearNPV genotypes, named HearSP1B+HearLB6, was co-occluded into virus occlusion bodies (OBs) in equal proportions [12], using previously established co-infection techniques [13]. The binary mixture of genotypes had insecticidal properties that were greater than those of the component genotypes, and this was the subject of a patent application for the development of a virus-based biological insecticide [12, 14].

As baculoviruses are obligate pathogens, these viruses can only be produced in larvae of susceptible species of insects (*in vivo*) or in cell culture systems (*in vitro*). However, *in vitro* production is expensive [15] and a number of technical issues limit its use as a method for the large-scale production of these viruses [16]. For example, in cell culture, virus populations rapidly accumulate defective particles with reduced insecticidal properties or reduced persistence on plant surfaces [17, 18, 19]. For this reason, all current baculovirus-based bioinsecticides are produced using *in vivo* systems, involving the infection of large cohorts of the host insect, or an alternative but susceptible host species. Given the limited host range of HearNPV, which is only infective for *Helicoverpa* spp. [20], the production of this virus is achieved using its homologous host, *H*. *armigera*.

*In vivo* production systems require optimization studies that take into account the biology and behavior of the host and the particular characteristics of each host-virus pathosystem. Factors such as quantity of inoculum, method and timing of inoculation, insect diet, rearing conditions, and virus harvesting have to be addressed [21]. These issues are usually established first at the laboratory scale and then suitably modified and standardized for pilot-plant or industrial scale production [21, 22, 23].

Like many species of Lepidoptera, *H*. *armigera* exhibits cannibalistic behavior during the larval stage [24]. Frequent cannibalism among larvae is undesirable in virus production systems since it adversely affects the quantity of occlusion bodies (OBs) produced in each container of larvae, or means that larvae have to be individualized post-inoculation, resulting in increases in the costs of OB production. Factors such as larval density or larvae stage directly influence the prevalence of cannibalism behavior in virus-infected insects [25, 26], even when there is no competition for food [27]. OB production is particularly affected by larval growth rate following inoculation, larval age at inoculation, inoculum concentration and incubation temperature [20]. Incubation temperature is relevant because it influences the rate of growth of the infected host and consequently the rate of replication of the virus, leading to a faster speed of kill [28]. As such, efficient OB production requires defining a balance between the conditions that result in maximum larval growth and a high incidence of virus-induced mortality in a short time period [23, 29]. In the present study, we examined how these factors can be optimized to improve the efficiency of production of HearSP1B:LB6 OBs in the homologous host, *H*. *armigera*.

## Materials and Methods

### Insect rearing and virus strain

A laboratory colony of *H*. *armigera* was maintained in the Universidad Pública de Navarra (UPNA) at 25±2°C, 70–80% relative humidity and 16:8 h day:night photoperiod on an artificial diet [30]. The colony was established with pupae received from the Centre for Ecology and Hydrology (CEH), Oxford, United Kingdom.

The virus used in this study was the co-occluded binary genotypic mixture HearSP1B:LB6 (in a 1:1 ratio), which was developed in a previous study and which possessed insecticidal properties better than those of individual component genotypes, as well as any other of the genotypes or genotype mixtures tested [12].

### Effects of larval stage and density on OB production

The effects of larval stage and density on OB production were evaluated in the final three larval instars, L_3_, L_4_ and L_5_, at rearing densities of 5, 10 and 20 larvae in 500 ml transparent plastic dishes (10 cm diameter at the base x 4 cm height) with a cardboard lid. Each dish contained a layer of artificial diet, 0.5 cm in depth, at the bottom of the dish [30]. In addition, 5 larvae of each stage were incubated individually in 30 ml transparent plastic cups (2.7 cm diameter at the base x 4.2 cm height), with a plastic cap. Newly molted larvae of each instar that had been starved for 12 hours were used. Larvae were inoculated with an OB suspension containing 100 mg/ml sucrose, 0.05 mg/ml Fluorella Blue food dye and the corresponding 90% lethal concentration (LC_90_) of OBs: 6.1 x 10^5^, 2.4 x 10^6^ and 2.5 x 10^7^ OBs/ml for L_3_, L_4_ and L_5_, respectively [31] using the droplet-feeding method [32]. As controls, identical numbers of larvae were inoculated with food dye and sucrose solution, without OBs. Larvae that drank the suspension in a 10 min period were transferred to the corresponding 500 ml plastic dishes for treatments involving densities of 5, 10 and 20 larvae/dish or 30 ml cups for individualized larvae. Dishes containing inoculated larvae were incubated at 26±1°C, 70±5°C relative humidity and darkness in a growth chamber. The numbers of larvae or infected corpses per dish were noted daily until all insects had either died or pupated. Insects showing signs of the final stages of polyhedrosis disease were individually transferred to Eppendorf tubes, incubated at for up to 6 h at 26°C until death and subsequently stored at -20°C. Pupae were discarded. The experiment was performed on three occasions.

For OB yield evaluation, larvae were thawed, individually homogenized in 1 ml of distilled water, and OBs were directly counted in triplicate using a Neubauer improved hemocytometer. The numbers of pupae, cannibalized larvae (larvae that disappeared or were partially devoured) and virus-killed larvae were averaged for each replicate and subjected to analysis of variance (ANOVA) and Tukey Test using the SPSS 15.0 program (IBM SPSS Statistics). Total OB production values per dish were obtained by the sum of the individual OBs/larva values. OBs/larva and total OB production per dish values were normalized by log transformation and subjected to ANOVA and Tukey Test (P<0.05) using the SPSS 15.0 program. The correlation between OB production and larval density was examined by Pearson coefficient as both variables were normally distributed.

### Effect of inoculation time, inoculum concentration and incubation temperature

To determine the optimal inoculation time for production of HearSP1B:LB6 OBs, groups of 24 larvae L_3_, L_4_ and L_5_ were inoculated at two intrastadial ages; as recently molted larvae (1–8 h post-molting) or as larvae at one day after molting (22–28 h post-molting). For this, larvae were weighed individually before inoculation and then allowed to consume a 90% lethal concentration (LC_90_) of OBs for each instar, using the droplet feeding method [31].

The influence of inoculum concentration on OB production was determined in groups of 24 recently molted (1–8 h post-molting) L_5_ larvae that had been inoculated with the LC_95_, LC_90_ and LC_80_, 1.5 x 10^8^, 2.5 x 10^7^ and 5.5 x 10^6^ OBs/ml, respectively [31]. Following inoculation, insects were individually transferred to 12-well plates containing artificial diet and incubated at 26±1°C, 70±5% relative humidity and darkness in a growth chamber until death or pupation. As controls, 24 larvae were allowed to drink the inoculation solution without OBs. The entire process was performed as previously described on three occasions. OB yields were determined as described above. As the prevalence of mortality varied with the inoculation time and viral concentration, OB yields were also estimated for groups of 100 inoculated larvae, in order to determine the total OB yield for each cohort of 100 inoculated larvae. Average OB production values (OBs/larva, OBs/mg larval weight and OBs per cohort of 100 inoculated larvae) were normalized by log transformation, whereas initial larval weight, cadaver weight and percentage of mortality values were normally distributed. All results were subjected to ANOVA and Tukey test using the SPSS 15.0 program. The correlations between larval weight at inoculation and cadaver weight, and also between log OB yield and cadaver weight were determined by examination of the Pearson coefficient, as all variables were normally distributed. The correlations between larval weight gain during the infection and the OB production per larva and per mg of larval weight were determined by examination of the Pearson coefficient and the Spearman coefficient.

To determine the effect of the incubation temperature on OB production, groups of 24 recently molted (1–8 h post-molting) L_5_ larvae were inoculated with the LC_95_ concentration of OBs using the droplet feeding technique. After inoculation, insects were individually transferred to 12-well plates containing artificial diet and were reared at 23±1°C, 26±1°C or 30±1°C in different incubation chambers in darkness, until they died of virus disease or pupated. Virus-induced mortality was recorded at intervals of 8 h. Moribund individuals showing the signs of lethal polyhedrosis disease were individually collected and triplicate samples of OBs were counted as described before. The experiment was performed five times. Time-mortality results were subjected to Weibull analysis using the GLIM program [33]. The validity of the Weibull model was determined using the Kaplan macro present in the GLIM program. OB counts were normalized by log transformation and subjected to ANOVA and Tukey test using the SPSS 15.0 program.

### Effect of incubation temperature on OB pathogenicity

The effect of incubation temperature on the pathogenicity of OBs produced at each temperature was estimated by concentration-mortality bioassays in *H*. *armigera* using the droplet feeding method. For this, groups of 24 recently molted (1–8 h post-molting) L_2_ larvae were inoculated with one of five different OB concentrations: 5.7 x 10^5^, 1.9 x 10^5^, 6.3 x 10^4^; 2.1 x 10^4^ and 7.0 x 10^3^ OBs/ml, which were previously found to result in between 95% and 5% mortality [31]. As controls, 24 larvae were inoculated with sucrose and food color solution without OBs. Following inoculation, insects were individually transferred to 24-well plates containing artificial diet and incubated at 26±1°C, 70±5% relative humidity in darkness in a growth chamber. Mortality was recorded at 24 h intervals during 10 days. The bioassay was performed three times. Concentration-mortality results were subjected to Probit analysis using the POLO-PC program [34]. A test for non-parallelism was performed on the regression slopes. Relative potencies were calculated as the ratio of effective concentrations relative to those of HearSP1B:LB6 produced at 23°C.

## Results

### Cannibalism

**Fig 1:**
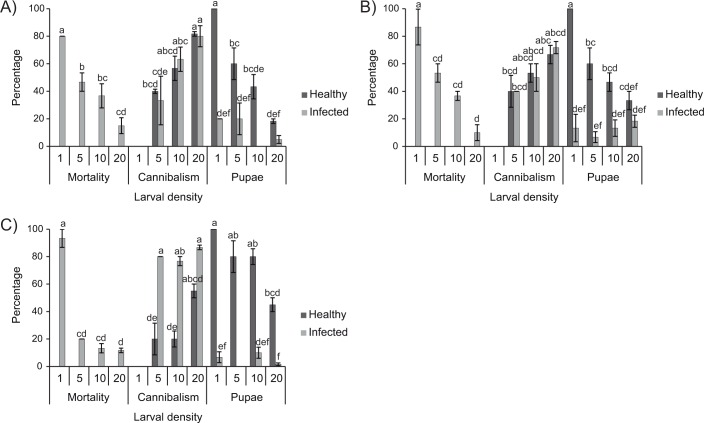
Percentage of larvae that pupated, virus-induced mortality and cannibalism. Percentages of larvae that pupated, or that died from lethal polyhedrosis or cannibalism were calculated in healthy and infected *H*. *armigera* larvae that consumed a LC_90_ concentration of HearSP1B:LB6 OBs, reared at densities of 1, 5, 10 and 20 larvae/dish. (A) L_3_, (B) L_4_, (C) L_5_. Values followed by identical letters did not differ significantly (ANOVA, Tukey test, P>0.05). Vertical bars indicate the standard error.

The prevalence of larval cannibalism was similar among the different instars evaluated, and similar in healthy and infected larvae (Tukey, P>0.05) (Fig 1), except in L_5_ in which cannibalism among infected larvae was significantly higher (77–87%) than among healthy larvae (20–55%) (Tukey, P<0.05) (Fig 1C). Cannibalism increased significantly with increasing larval density (F_3,71_ = 57.12, P<0.001). In L_3_ and L_4_ instars, the effect of larval density was similar in infected and healthy larvae, with cannibalism increasing from ~40% at the density of 5 larvae/dish to ~80% at a density of 20 larvae/dish (Fig 1A and 1B). In contrast, in L_5_ cannibalism varied from 20–55% in healthy larvae compared to 80–87% in infected larvae across all densities (Tukey, P>0.05) (Fig 1C).

**Fig 2:**
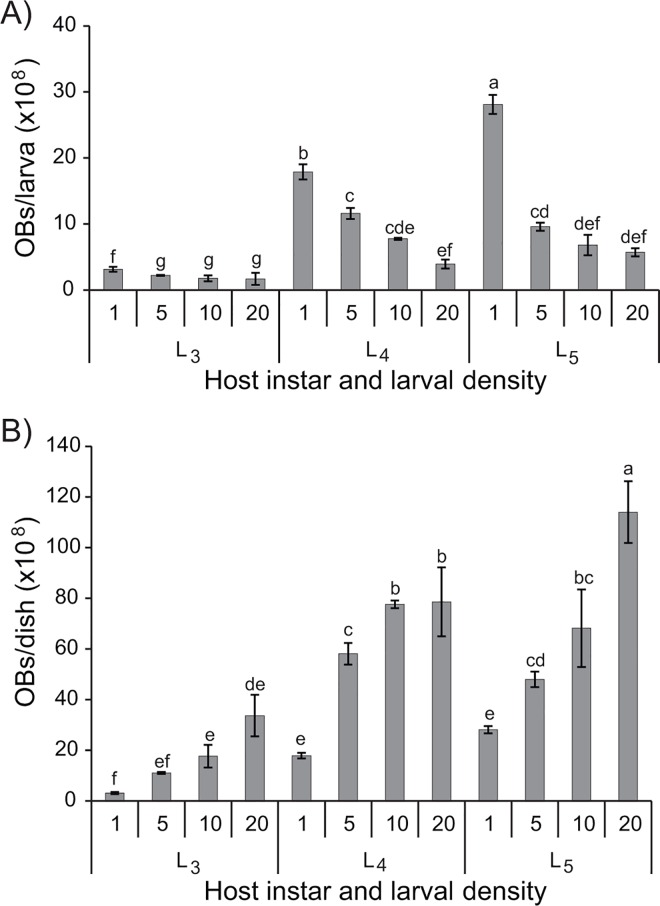
Mean OB production in L_3_, L_4_ and L_5_ *H*. *armigera* larvae. Larvae were inoculated with a LC_90_ concentration of HearSP1B:LB6 OBs and reared at densities of 1, 5, 10 and 20 larvae/dish. (A) Production expressed as OBs/larva, and (B) Total OB production per dish. Values followed by identical letters did not differ significantly (ANOVA, Tukey test, P>0.05). Vertical bars indicate the standard error.

Cannibalism directly influenced both the average numbers of virus-killed larvae and the final OB yields per larva and per dish. The percentage of virus-induced mortality (80–93%) was significantly higher in individualized larvae compared to those incubated at higher densities (12–53%) (F_3,71_ = 6.05, P<0.001) (Fig 1). OB production, either OBs/larva or OBs/dish (not including larvae that pupated or that died from cannibalism), was negatively correlated with larval density in each of the three instars tested (Pearson, r = -0.97 for L_3_, r = -0.99 for L_4_ and r = -0.99 for L_5_) (Fig 2A). This effect was most clearly observed in L_4_ and L_5_ in which the production of OBs decreased progressively from 1.8 x 10^9^ and 2.8 x 10^9^ OBs/larvae in individualized larvae, respectively, to 1.2 x 10^9^ and 5.7 x 10^8^ OBs/larvae, respectively, in larvae reared at the highest density (Tukey, P<0.05).

The total production of OBs among all the virus-killed larvae from each dish, representing the product of the mean number of OBs per larva and the average number of virus-killed larvae per dish, not including larvae that pupated or that died from cannibalism, varied significantly with the instar (F_2,35_ = 10.13, P<0.001) and density (F_3,35_ = 6.80, P<0.001) (Fig 2B). However, despite being seeded with 20 larvae per dish, the highest density only produced 10.7-fold more OBs/dish in L_3_, 4.4-fold more OBs/dish in L_4_ and 3.9-fold more OBs/dish in L_5_, compared to the OB production observed in individualized larvae (Fig 2B). Overall, these results led to the decision to produce HearSP1B:LB6 OBs in individualized larvae.

### Selection of inoculation time

**Fig 3:**
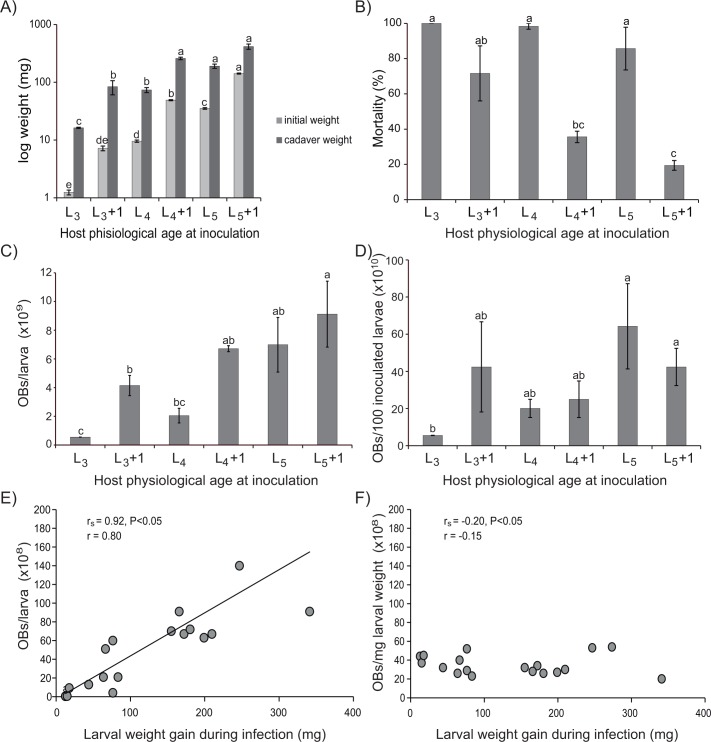
Parameters observed to determine the optimum inoculation time. (A) Initial and cadaver weight of larvae, (B) Percentage of virus-induced larval mortality, (C) Production of OBs/larva, (D) OB production expressed as OBs per cohort of 100 larvae, (E) Production of OBs/larva versus larval weight gain during infection, and (F) Production of OBs/mg of larva versus larval weight gain during infection in insects inoculated with a LC_90_ concentration of HearSP1B:LB6 OBs as recently molted larvae (L_3_, L_4_, L_5_) and at one day after molting to (L_3_+1, L_4_+1, L_5_+1). Values followed by identical letters did not differ significantly (ANOVA, Tukey test, P>0.05). Vertical bars indicate the standard error.

The initial larval weight varied significantly among larvae inoculated at different intrastadial ages and stages (F_5,17_ = 1637.4, P<0.001) (Fig 3A). All instars showed an ~80% increase in body weight in the period between molting and when weighed at 1 day post-molting (Tukey, P>0.05). A positive correlation was observed between the initial larval weight and the cadaver weight (Pearson r = 0.96). The highest weights were observed in the cadavers of late instar larvae (L_4_+1, L_5_, L_5_+1) that were significantly heavier than the cadavers of insects inoculated at earlier instars (F_5,17_ = 75.1, P<0.001) (Fig 3A). Within the same instar, larvae inoculated one day after molting died at a consistently higher body weight than larvae inoculated when recently molted (Tukey, P<0.05) (Fig 3A).

The intrastadial age at the moment of inoculation had a marked effect on the percentage of larval mortality (F_5,17_ = 15.1, P<0.001) (Fig 3B). In recently molted larvae 85–100% of lethal polyhedrosis was observed across all instars tested, in line with the high OB concentrations present in the inocula. However, larvae inoculated one day after molting presented significantly lower percentages of mortality (21–72%). Moreover, the difference between the expected and the observed mortalities increased significantly with increasing instar (Tukey, P<0.05), so that the mortalities of L_3_, L_4_ and L_5_ larvae inoculated one day after molting were 72, 36 and 21%, respectively.

As observed in the previous experiment, OB production increased significantly with instar (F_5,17_ = 15.1, P<0.001), and a positive correlation was observed between OB production and cadaver weight (Pearson r = 0.92). The highest numbers of OBs were produced in larvae inoculated one day after molting to L_4_, recently molted L_5_, and one day after molting to L_5_ (6.7–9.1 x 10^9^ OBs/larva) (Tukey, P<0.05) (Fig 3C). However, due to the differences obtained in the percentage of virus-induced mortality in larvae inoculated at different intrastadial ages and stages, which ranged from 19.5 to 100%, the total OB yield per cohort of 100 inoculated larvae differed significantly with inoculation intrastadial age and stage (F_5,17_ = 4.6, P = 0.01) (Fig 3D). The most productive treatment was newly molted L_5_, with a total production of 6.0 x 10^11^ OBs per 100 inoculated larvae (Fig 3D). A positive correlation was observed between the larval weight gain during the infection and the OB production per larva (Pearson r = 0.80; Spearman r_s_ = 0.92, P<0.05) (Fig 3E), whereas no correlation was observed between the larval weight gain during the infection and the OB production per mg of larval weight (Pearson r = -0.20; Spearman r_s_ = -0.15, P>0.05) (Fig 3F). According to these results, the maximum HearSP1B:LB6 OB production was achieved by inoculation of recently molted L_5_ larvae.

### Selection of inoculum concentration

**Fig 4:**
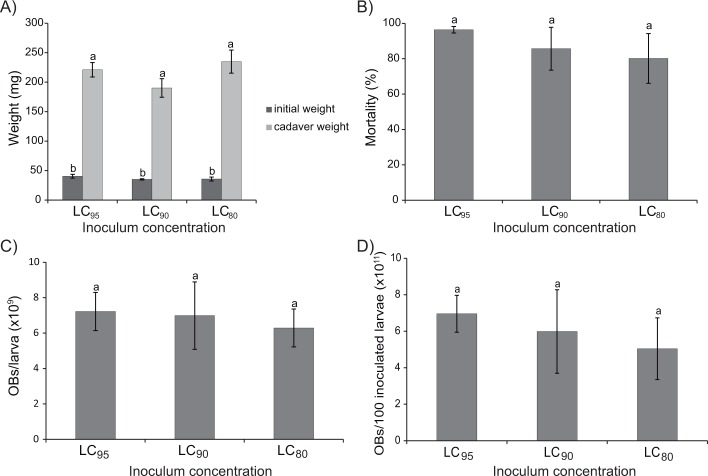
Parameters observed to determine the optimum inoculum concentration. (A) Initial and virus-killed cadaver weight, (B) Percentage of larval mortality, (C) Production of OBs/larva, and (D) OB production per cohort of 100 inoculated larvae, in recently molted L_5_ *H*. *armigera* inoculated with LC_95_, LC_90_ and LC_80_ concentrations of HearSP1B:LB6 OBs. Values followed by identical letters did not differ significantly (ANOVA and Tukey test, P>0.05). Vertical bars indicate the standard error.

Inoculum concentration did not significantly affect body weight at death which ranged from an average of 180 to 232 mg (F_2,6_ = 1.2, P = 0.13) (Fig 4A), or the percentage of mortality (80.1–96.4%) (F_2,6_ = 0.6, P = 0.58) (Fig 4B). The different inoculum concentrations tested also produced similar OB yields (Fig 4C). For example, newly molted L_5_ produced between 6.3 x 10^9^ and 7.2 x 10^9^ OBs/larva (F_2,6_ = 0.1, P = 0.90) (Fig 4C) and between 5.3 x 10^11^ and 6.9 x 10^11^ OBs for each cohort of 100 inoculated larvae (F_2,6_ = 0.3, P = 0.77) (Fig 4D).

### Incubation temperature

**Fig 5:**
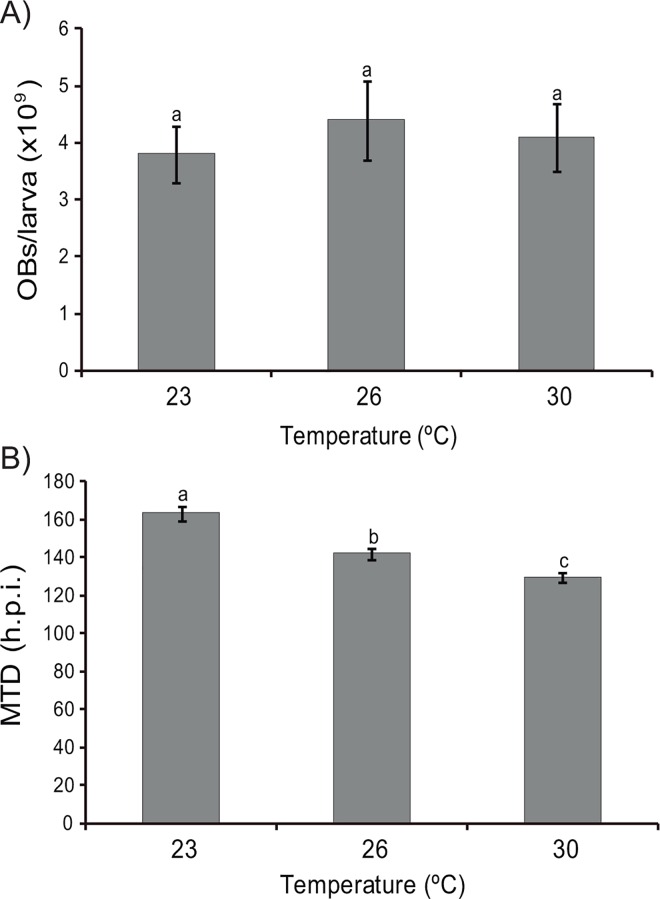
Parameters observed to determine the optimum incubation temperature. (A) Mean OB production (OBs/larva) and (B) Mean time to death (hours post-infection) of L_5_ *H*. *armigera* larvae after inoculation with a LC_95_ concentration of HearSP1B:LB6 OBs and incubated at 23, 26 and 30°C until death. Values followed by identical letters did not differ significantly (OB production: ANOVA, Tukey test, P>0.05; Mean time to death: Weibull analysis, P<0.05). Vertical bars indicate the standard error.

Incubation temperature did not significantly affect the prevalence of larval mortality (F_2,12_ = 1.5, P = 0.52). Mortality of L_5_ incubated at 23, 26 and 30°C was 88, 91 and 86%, respectively. Similarly, final OB yields (3.2–4.2 x 10^9^ OBs/larva) did not differ significantly in cadavers that had been incubated at different temperatures (F_2,12_ = 0.3, P = 0.75) (Fig 5A). However, insects incubated at 30°C died significantly more rapidly than larvae incubated at 26°C and 23°C, respectively (Fig 5B).

Finally, incubation temperature did not significantly influence the insecticidal properties of the OBs (Table 1). The pathogenicity of OBs recovered from larvae maintained at 23, 26 and 30°C were very similar with LC_50_ values between 1.1 x 10^4^ and 1.8 x 10^4^ OBs/ml when bioassayed in L_2_ larvae. Therefore, the 30°C incubation temperature was selected as the optimal temperature for the efficient production of HearSP1B:LB6 OBs in *H*. *armigera*, as L_5_ larvae produced similar quantities of OBs, and OBs of similar pathogenicity, but died faster than conspecifics incubated at lower temperatures.

**Table 1:** Lethal concentration (LC_50_) values and relative potencies of HearSP1B:LB6 OBs produced in L_5_ *Helicoverpa armigera* larvae incubated at different temperatures. OBs were bioassayed in second instar larvae.

Temperature	LC_50_	Relative	Fiducial limits 95%	
	(OBs/ml)	potency	Low	High
23°C	1.5 x 10^4^	1	-	-
26°C	1.1 x 10^4^	1.3	0.8	2.0
30°C	1.8 x 10^4^	0.8	0.5	1.3

Logit regressions were fitted in POLO-PC. A test for non-parallelism was not significant (χ^2^ = 5.7, df = 2, P>0.05), such that regressions were fitted with a common slope of 1.13 ± 0.15 (mean ± S.E.). Relative potencies were calculated as the ratio of effective concentrations relative to those of HearSP1B:LB6 produced at 23°C.

## Discussion

Large scale OB production for use in biological insecticide products has to be performed in permissive species of insects, in this case *H*. *armigera* larvae. However, *H*. *armigera* can show cannibalistic habits during the larval stage. The prevalence of cannibalism observed in this study was very high in all three instars tested and increased markedly with larval density. Furthermore, in the fifth instar, cannibalism was significantly higher among infected larvae than among healthy larvae, whereas in L_3_ and L_4_, infection status had no significant effect on cannibalism. A high prevalence of cannibalism towards larvae infected by entomopathogenic viruses has been attributed in previous studies to lower mobility and sluggish responses of diseased larvae, making diseased insects more likely to be victims of conspecific predation than healthy insects [24, 35, 36]. Mortality due to cannibalism in *H*. *armigera* was similar among the different larval stages, as observed in a previous study on *H*. *zea* [37]. In contrast, studies performed in other insect species have frequently observed that cannibalism is stage dependent, with a greater propensity for intraspecific predation in the later instars [25, 35, 38, 39], or in the penultimate instar [26]. In addition to a range of evolutionary advantages [39], cannibalism behavior can favor the survival of more robust and fecund individuals in laboratory colonies [27]. However, cannibalism is not desirable during OB production procedures as the total number of larvae is reduced, which reduces the overall yield of OB produced in each cohort of insects [25, 40]. For these reasons, individualized rearing of *H*. *armigera* larvae post-inoculation appears to be necessary for efficient production of HearSP1B:LB6 OBs, despite the fact that individualized rearing implies additional labor and materials costs. For example, handling of individualized larvae involves approximately three times longer than handling of groups of larvae. Additionally, as each larva requires an individual plastic cup, a lid and a piece of diet, individualized rearing is likely to increase the total cost of rearing materials. However, the highest density of 20 larvae/dish resulted in increases of just 10.7, 4.4 and 3.9-fold in the overall production of OBs in L_3_, L_4_ and L_5_, respectively, than individualized larvae. Therefore, the greater production of OBs per individualized larva is therefore likely to overcome the additional costs of individualized rearing.

OB production, measured in terms of OBs per larva, increased with increasing larval stage and age at inoculation time, which was positively correlated with the larval weight at inoculation and cadaver weight. Thus, larvae inoculated one day after molting to L_4_, recently molted L_5_, and L_5_ at one day after molting, and were the most productive developmental states. Previous studies have reported a direct relationship between larval age and OB production in *H*. *armigera*. In the present study, *H*. *armigera* larvae inoculated at later instars yielded between 6.7 x 10^9^ and 9.1 x 10^9^ OBs/larva, which is comparable to the yields observed in *H*. *armigera* late instars reported previously (1.7 x 10^9^−1.2 x 10^10^ OBs/larva) [41, 42, 43]. However, mortality was markedly higher in recently molted larvae than in larvae inoculated one day after molting, indicating that recently molted larvae are more susceptible to baculovirus infection than older conspecifics, as previously reported in other host-baculovirus pathosystems [44, 45]. Larvae infected 24 h after moulting show intrastadial development resistance (IDR) compared to larvae inoculated immediately after moulting. This is likely the result of several anti-viral defenses, including the sloughing of infected midgut cells before the virus spreads beyond the midgut or hormonally-mediated defenses [44, 45]. The inoculum concentration needed to achieve more than 80% mortality in L_5_ one day after molting must be therefore substantially increased compared to larval inoculated immediately following molting. Moreover, recently molted larvae are easier to handle, as larvae are selected at pre-molting and starved until molting, whereas those inoculated one day after molting need to be previously selected as newly molted, reared on diet during 18 h, and removed from diet 8 h prior to inoculation. Therefore, recently molted L_5_ was selected as the most suitable inoculation stage for the production of HearSP1B:LB6 OBs.

The quantity of OB inoculum consumed by larvae can have an important effect on the production of OBs in each larva since high doses of OBs can hasten the death of the larva, resulting in reduced weight gain during the period of infection and consequently fewer OBs produced in each insect [42, 46]. However, inoculum OB concentrations tested in the present study resulted in a similar prevalence of mortality and similar OB yields from experimental insects. OB production per mg of larval body weight did not vary significantly among the different inoculum concentrations, but was clearly affected by larval weight. In fact, we observed a positive correlation between the larval weight gain during the infection and the OB production per larva, which is in agreement with previous studies performed with *L*. *dispar* inoculated with its homologous NPV [22]. However, no correlation was observed between the larval weight gain during the infection and the OB production per mg of larval weight. Previous studies on OB production in third instar *H*. *armigera* infected by a Chinese isolate of HearNPV reported a mean value of 6.0 x 10^9^ OBs/larva, which increased to 1.0 x 10^10^ OBs/larva in the fifth instar [47]. However, Sun et al. [47] did not detect significant differences in OB production/mg of larval weight, which was approximately 3 x 10^7^ OBs/mg in all instars tested, clearly within the range of 2.0 x 10^7^–5.0 x 10^7^ OBs/mg of larval weight observed in the present study. Our observation that the three inoculum concentrations tested resulted in similar OB yields is likely due to the fact that inoculated larvae reached similar final weights at the moment of death.

OB production may also be influenced by incubation temperature, which directly affects the larval growth rate and the rate of virus replication [29]. At higher temperatures larvae feed and grow faster, and cell metabolism is accelerated resulting in faster virus replication, which can lead to premature host death and reduced OB yields compared to insects reared at lower temperatures. Mehrvar et al. [43] obtained an almost 1.5-fold increase in OB yield by incubating *H*. *armigera* infected larvae at 25°C rather than at 30°C. Studies performed with other species, such as *S*. *litura* [29], *Lymantria dispar* [22] or *Mamestra brassicae* [48] have consistently reported the highest OB production in larvae reared in the range 25–30°C following inoculation, which reflects the optimum temperature range for the growth of the host insects. In the present study rearing at 30°C accelerated death by 13 or 34 h compared to larvae reared at 26 or 23°C, respectively. Similar effects have been reported in other species, including *Anticarsia gemmatalis* [28] *S*. *litura* [29], *Diatraea saccharalis* inoculated with heterologous NPVs [49], and *Trichoplusia ni* inoculated with *Autographa californica* MNPV [50]. Furthermore, high incubation temperatures may affect the insecticidal properties of OBs, particularly by favoring the propagation of bacterial contaminants [51], which contribute to OB degradation following the death of the insect host [29]. However in the present study, incubation temperature did not affect the biological activity of HearSP1B:LB6 OBs in terms of concentration-mortality metrics, which agrees with previous studies performed on *S*. *litura* infected with its homologous NPV [29], and *L*. *dispar* infected with LdMNPV [22].

Considering the results obtained in this study, we conclude that efficient production of the HearSP1B:LB6 co-occluded mixture of OBs should be performed by inoculation of recently molted L_5_ with an LC_80_ concentration (5.5 x 10^6^ OBs/ml) of inoculum followed by incubation of individualized larvae at 30°C. Using this system it is possible to produce large quantities of OBs suitable for use as an effective biological insecticide for control of this pest.
